# Optimization of Process Parameters in CNC Turning of Aluminum 7075 Alloy Using L27 Array-Based Taguchi Method

**DOI:** 10.3390/ma14164470

**Published:** 2021-08-10

**Authors:** Mohammad Nishat Akhtar, T. Sathish, V. Mohanavel, Asif Afzal, K. Arul, M. Ravichandran, Inzarulfaisham Abd Rahim, S. S. N. Alhady, Elmi Abu Bakar, B. Saleh

**Affiliations:** 1 School of Aerospace Engineering, Universiti Sains Malaysia, Nibong Tebal 14300, Penang, Malaysia; nishat@usm.my; 2 Department of Mechanical Engineering, Saveetha School of Engineering, SIMATS, Chennai 602105, Tamil Nadu, India; sathish.sailer@gmail.com; 3 Centre for Materials Engineering and Regenerative Medicine, Bharath Institute of Higher Education and Research, Chennai 600073, Tamil Nadu, India; mohanavel2k16@gmail.com; 4 Department of Mechanical Engineering, P. A. College of Engineering, Visvesvaraya Technological University, Belagavi 574153, Mangaluru, India; asif.afzal86@gmail.com; 5 Department of Mechanical Engineering, Jeppiaar SRR Engineering College, Chennai 603103, Tamil Nadu, India; arul.phd2014@gmail.com; 6 Department of Mechanical Engineering, K Ramakrishnan College of Engineering, Trichy 621 112, Tamil Nadu, India; smravichandran@hotmail.com; 7 School of Mechanical Engineering, Universiti Sains Malaysia, Nibong Tebal 14300, Penang, Malaysia; inzarul@usm.my; 8 School of Electrical and Electronic Engineering, Universiti Sains Malaysia, Nibong Tebal 14300, Penang, Malaysia; 9 Mechanical Engineering Department, College of Engineering, Taif University, P.O. Box 11099, Taif 21944, Saudi Arabia; b.saleh@tu.edu.sa

**Keywords:** Al7075, turning parameters, Taguchi method, L27 array, signal to noise ratio

## Abstract

With the advent of the industrial revolution 4.0, the goal of the manufacturing industry is to produce a large number of products in relatively less time. This study applies the Taguchi L27 orthogonal array methodological paradigm along with response surface design. This work optimizes the process parameters in the turning of Aluminum Alloy 7075 using a Computer Numerical Control (CNC) machine. The optimal parameters influenced the rate of metal removal, the roughness of the machined surface, and the force of cutting. This experimental investigation deals with the optimization of speed (800 rpm, 1200 rpm, and 1600 rpm) and feed (0.15, 0.20, and 0.25 mm/rev) in addition to cutting depth (1.0, 1.5, and 2.0 mm) on the turning of Aluminum 7075 alloy in a CNC machine. The outcome in terms of results such as the removal rate of material (maximum), roughness on the machined surface (minimum), along with cutting force (least amount) were improved by the L27 array Taguchi method. There were 27 specimens of Al7075 alloy produced as per the array, and the corresponding responses were measured with the help of various direct contact and indirect contact sensors. Results were concluded all the way through diagrams of main effects in favor of signal-to-noise ratios and diagrams of surfaces with contour diagrams for various combinations of responses.

## 1. Introduction and Background

In the current market competition, industrial sectors are focusing on high production with minimum cost. Especially, manufacturing industries are concentrating on a variety of products in batch production. Nowadays, composites have found a wider use of materials in most of the top leading manufacturing sectors. Machining is the essential choice to produce the components with closer tolerances. The second generation of hybrid composites is found in many engineering fields and acts as a better alternative for numerous materials. Specifically, automotive, aircraft, and locomotive industries need to replace ferrous materials in mechanical components with lighter high strength aluminum (Al) matrix composites. The machining process becomes very important, and it is always accomplished to convert the composites into engineering components [1]. Metal Matrix Composites (MMCs) are not easy to machine, due to the hardness and abrasive nature of the reinforcing particles. Thus, the machinability studies have acquired greater importance in the area of composites [2]. The researchers have considered numerous aspects such as the metallurgical characteristics, geometry of the cutting tool, work piece characteristics, and process parameters such as cutting speed, feed rate, and depth of cut. The cutting zone temperature, surface roughness, and tool wear are the significant output responses that were influenced by the factors such as chemical, physical, and thermomechanical parameters that interact during the machining operation [3]. The effect of cutting speed, feed, depth of cut, and work piece with different hardness values of cutting forces during the machining of AISI H11 steel is considered. They developed a mathematical model using RSM and validated it using Analysis of Variance (ANOVA) software [4]. The optimum finding of output responses in the turning process and the selection of parameters along with their levels were achieved through using the Taguchi method [5]. The design and analysis of experiments used the Taguchi method as a methodical paradigm. The authors demonstrated the triumphant method to create an excellent improvement of products with low cost [6]. Managing several characteristics of performance through the Taguchi method requires referencing various research studies [7]. Various researchers’ articles gave thorough explanations concerning the significance of measures for turning through the Taguchi method by means of a variety of constraints [8]. An investigation of laser micromachining on Al 7075 for optimization provided the basic details and properties about Al 7075 in a clear manner such as chemical compositions and mechanical properties in a tabulated form [9]. Optimum finding of SR and CF through an AA7039/Al_2_O_3_-based composite metal matrix was achieved by means of the Taguchi method with neural networks [10]. One study examined machining on Al7075-T7351 by experimentation and mentioned that there was a physically powerful correlation among the thickness of the chip and roughness of the surface [11]. An experimental study of three hybrid composites of Al7075 material used the turning process and focused on the details about force of cutting for the various considerations of speed of cutting (N), rate of feed (F), and cutting depth (D) [12]. Researchers gave an explanation regarding the preparation of experimentation by relying on an orthogonal array (L27) to put forward the method of Taguchi to decide essential parameters that can considerably impact on the surface roughness and force of cutting [13]. They investigated the turning process through speed ranging from 125 to 185 m/min, feed ranging from 0.12 to 0.20 mm/min, Depth of Cut (DOC) ranging from 0.5 to 0.8 mm, and ratio of cutting fluid ranging from 4.0% to 12.0% with the Taguchi gray relational method. From this study, researchers were able to draw conclusions about the DOC, speed of cutting, ratios of mixture of the cutting fluid, and rate of feed. Amongst all factors, the DOC was found to be most the influencing factor [14]. An orthogonal array (L9) was applied in the Taguchi method using an experimental examination on tool life, force of cutting, roughness of surface, and responses by considering parameters, i.e., speed of cutting (N) and feed (F) with DOC. The optimization process was effectively utilized in their research, which presented the features of the parameters’ correlations as well as the outcome results [15]. A turning process with cutting speed starting from 135 to 285 m/min, rate of feed starting from 0.08 to 0.32 mm/min, and DOC from 0.6 to 1.6 mm was used for inspecting tool life. Force of cutting with roughness on a machined surface through the Taguchi method was an addition for gray relational analysis. Nonetheless, they gave an explanation of the minute procedure regarding their investigation and projected the values of optimization for the most excellent production on the turning process. They evidently reported that the feed rate generates a superior contribution amongst the other factors considered [16]. In the turning process, the material of AISI (American Iron and Steel Institute)-1016 carbon steels was turned by cubic boron nitride with the consideration of different parameters. The specs of the parameters were speed from 360 to 1150 m/min, feed from 0.05 to 0.13 mm/min, and DOC from 0.5 to 1.0 mm from beginning to end. The Taguchi technique was taken for the evaluation of surface roughness. They noted that 64% of the main contribution order was due to the rate of feed, speed, and DOC [17]. Investigations were made on the turning of AISI 1045 steel under the dry cutting process through the Taguchi method with a variety of machining parameters such as speed ranging from 160 to 620 m/min, feed ranging from 0.3 to 0.5 mm/min, and DOC ranging from 0.7 to 0.9 mm. They had completed the computation of signal-to-noise ratios by using a method for roughness on the machined surface and the rate of metal removed manually and provided a match through the experiment [18]. In an experimental study of CNC operation on aluminum, the operating cutting speed ranged from 600 to 1000 m/min, the feed rate ranged from 0.1 up to 0.2 mm/min, and the DOC ranged from 0.5 to 1.5 mm for MRR through Taguchi method analysis [19]. Discussion was made of the specifications of turning Al alloy through a CNC machine with the speed of cutting ranging from 600 to 700 m/min, rate of feed ranging from 25 to 50 mm/min, and DOC ranging from 0.2 to 0.4 mm. They also pointed out the limitations that had an effect on surface roughness through a Fishbone diagram [20]. The effects of parameters using ANN (Artificial Neural Networks) with a computation based on tool life during machining established a high-quality correlation among the obtained theoretical and experimental values [21,22,23]. Although the experimental investigation and modeling of Al7075 alloy was investigated by many researchers, no work was found on the modeling of CNC turning of Al7075 alloy using response surface methodology (RSM). So, in the proposed work, RSM has been used for modeling CNC turning of Al7075 alloy using the L27 Taguchi method.

## 2. Experimental Arrangement and Methodology

**Figure 1:**
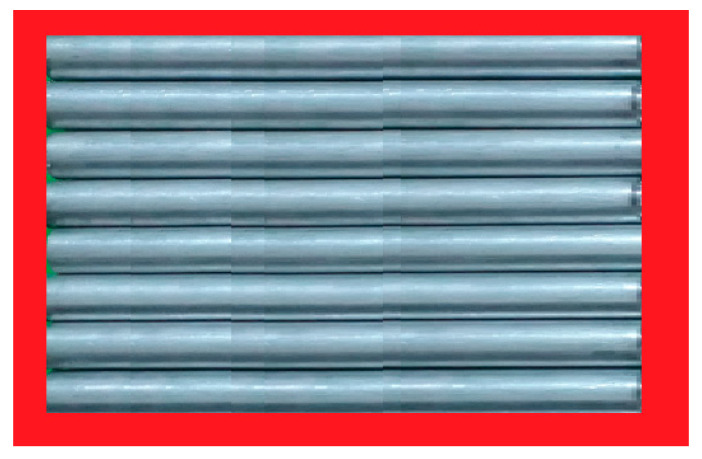
Raw materials of Aluminum alloy Al7075 rods in multiple numbers.

Aluminum alloy AA7075 with 24 mm diameter rods were procured; then, the purchased materials were visually tested regarding any cracks or damage on the surface of the materials. Each specimen was prepared with a length of 150 mm and diameter of 24 mm. There is no change in diameter for the raw materials, and only the length needed to be cut. So, the long raw material was cut as 150 mm length rods. There were more than 27 raw specimens, which were prepared from the purchased material for turning, as shown in Figure 1. 

**Figure 2:**
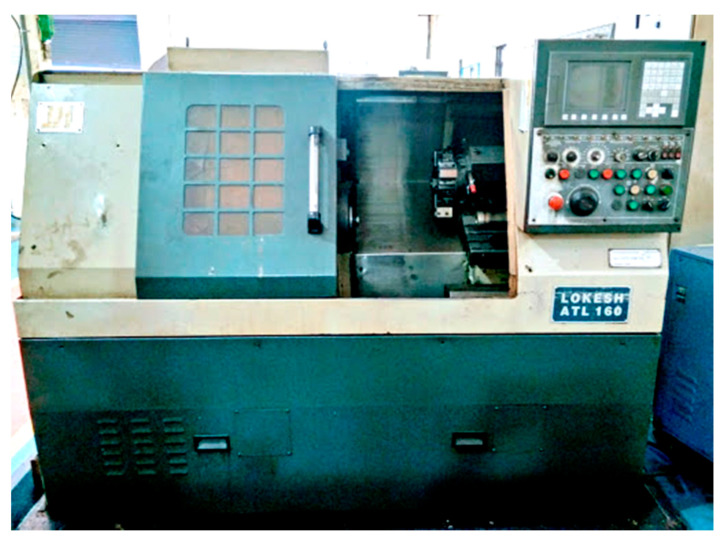
CNC machine used for the experimental investigation.

The turning operation was considered for this investigation. This turning can be done by various machines such as normal lathes, etc., but the greater accuracy can be obtained from CNC machine only [24]. So, in this investigation, turning operations are perfectly completed with the help of a FANUC Series 0i controlled CNC machine. Figure 2 shows the CNC machine chosen for the experiments and the corresponding most preferable specification of that CNC machine, including the model of the machine, the spindle power, and size of the chuck; the spindle bore dimensions are clearly mentioned in Table 1.

**Figure 3:**
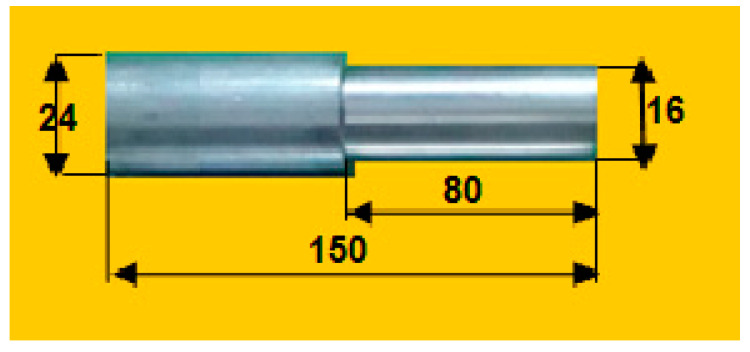
Turning operation: image after turning of Al7075 alloy with its dimensions.

Table 1 also mentions the turning process parameters (N, F, and D) and three responses (MRR, SR, and CF) for investigational experiments. The raw material is turned by using a CNC machine as per the dimensions shown in Figure 3, i.e., 80 mm long, 16 mm diameter turned from the 24 mm diameter Al7075 rod. In the CNC turning process, the heat was generated in between the specimens and tool; that heat was carried away from both of them by applying coolant such as soluble oil. 

Using an L27 orthogonal array, the twenty-seven numbers of specimens were machined by applying 27 runs with correlations of parameters in CNC turning. The corresponding individual specimen responses such as cutting forces were measured with the help of a lathe tool dynamometer fixed on the machining setup in the CNC machine. The sensing elements have higher accuracy by using a strain gauge. The surface roughness was measured with the help of a surface roughness tester (Model: Mitutoyo SJ210) with a measuring range up to 160 microns. The evaluation parameters of this instrument such as Rx, Ry, Rz, and cut-off length were specified as 0.08, 0.25, 0.8, and 2.5 mm, and the sampling length of the instrument was specified as 0.08, 0.25, 0.8, and 2.5 mm. This instrument is used extremely often in industry with different sampling lengths (0.3 to 16.0 mm with 0.01 mm interval). All the responses compared chose optimum process parameters through the assistance of the Taguchi investigation method. Comparisons were completed as per the ideas shown in Table 2. In the turning process, MRR was preferred to be set as large (high), and a smaller amount of SR was favored for machining. Similarly, the minimum CF was set to be suitable for the operations [25].

## 3. Assessment of Results Using Taguchi Analysis and RSM

**Figure 4:**
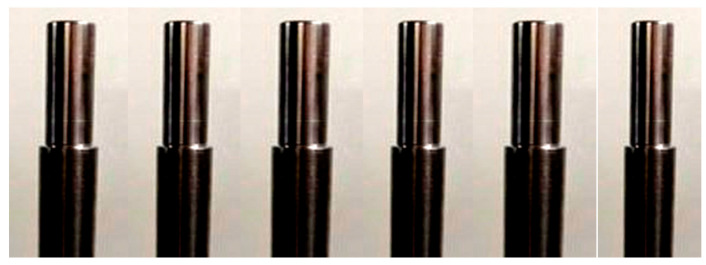
Sample specimens after turning with respect to process parameters.

There were 27 specimens that were turned as per the dimensions mentioned in Figure 3 with the conditions N, F, and D, as shown in Table 3. After completing the tuning process, the specimens were fully cleaned, and oil was applied on the surface. Then, the experimentally measured responses of an individual specimen’s results based on an L27 orthogonal array of process parameters were measured with the help Minitab 17 statistical analysis on the CNC machine, as mentioned in Table 3. Figure 4 shows the sample specimen after turning with respect to process parameters. It is to be noted that excess raw specimens were kept as balance.

**Figure 5:**
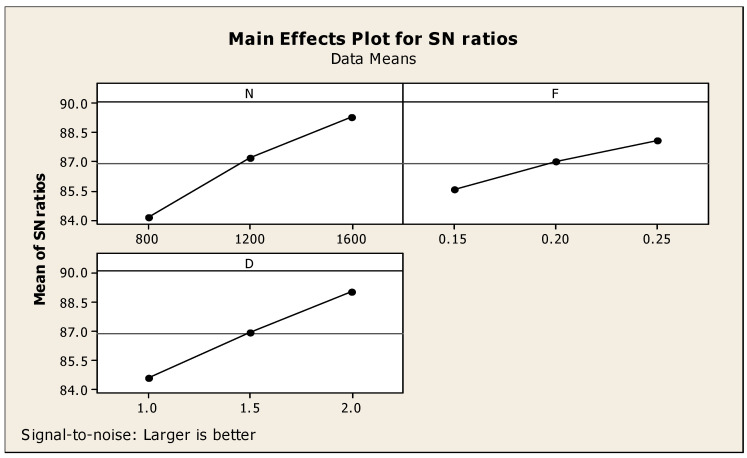
MRR-related major effects diagram with respect to signal to noise ratios.

**Figure 6:**
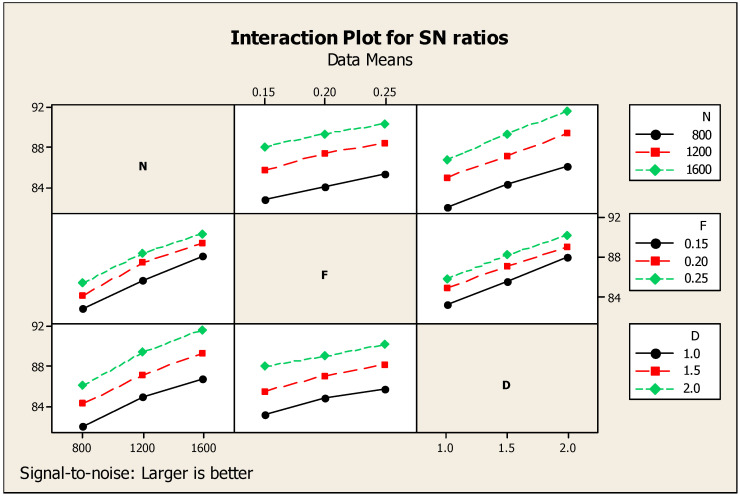
MRR interaction diagram.

For the initial analysis, only one response—that is, MRR–was taken for the consideration. Table 4 clearly mentions the Taguchi analysis details of MRR versus speed (N), feed (F), and depth of cut (D). In addition, it also mentions the designated rank: i.e., rank one for feed, second rank for depth of cut, and third rank for speed. The corresponding main effects diagram regarding the ratio of signal to noise for the material removal rate is highlighted in Figure 5 under the larger is better condition [26]. The highest MRR is obtained at the conditions of 1600 rpm cutting speed, 0.25 mm per min of feed, and 2.0 mm of cutting depth as shown in Figure 5 with respect to the signal-to-noise (SN) ratio. The interaction diagram regarding the SN ratios of MRR is shown in Figure 6. It also provides the details of the individual factors such as N, F, and D as the separate diagrams of the combination with the response such as MRR. 

**Figure 7:**
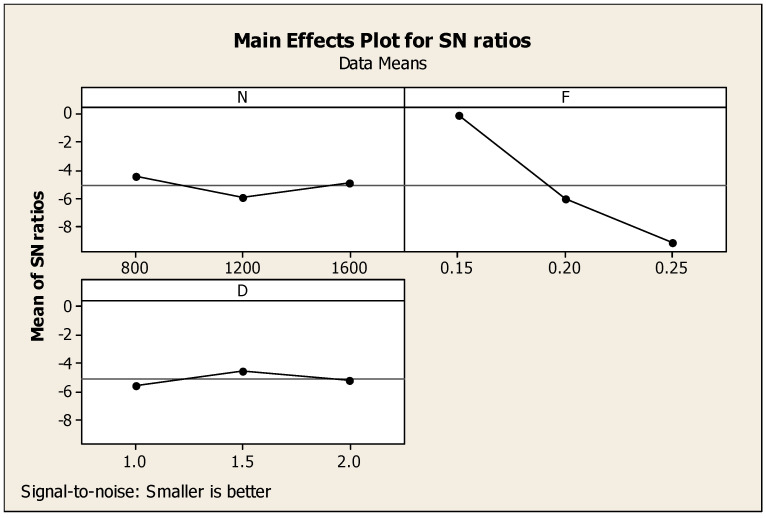
SR major effects diagram.

**Figure 8:**
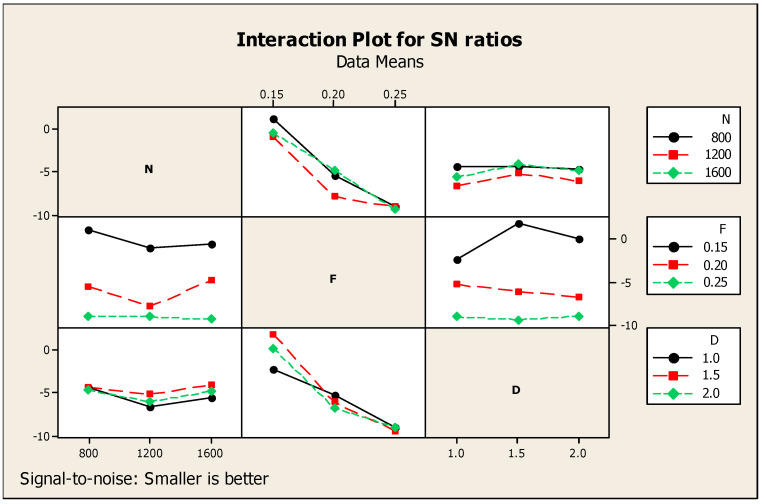
SR diagram for interaction.

With respect to correlation and the specs of MRR and speed, only the roughness on the machined surface is taken as a single response. Table 5 shows the Taguchi analysis details of SR versus N, F, and D and designated the first rank for feed, second rank for speed, and third rank for the depth of cut. Figure 7 correlates the ratio of signal to noise of the roughness on the machined surface. In addition, the corresponding interaction diagram with respect to the influencing factors with the single response for the ratio of signal to noise of SR is shown in Figure 8 under the smaller is better condition. It also offers the information of the factors such as N, F, and D as the part diagrams of the combination with the response such as SR. In accordance with the minimum condition, SR specs were formalized as 1200 rpm cutting speed, 0.25 mm per min feed rate, and one mm cutting depth, as highlighted in Figure 7 with regard to the signal-to-noise ratio (SNR).

**Figure 9:**
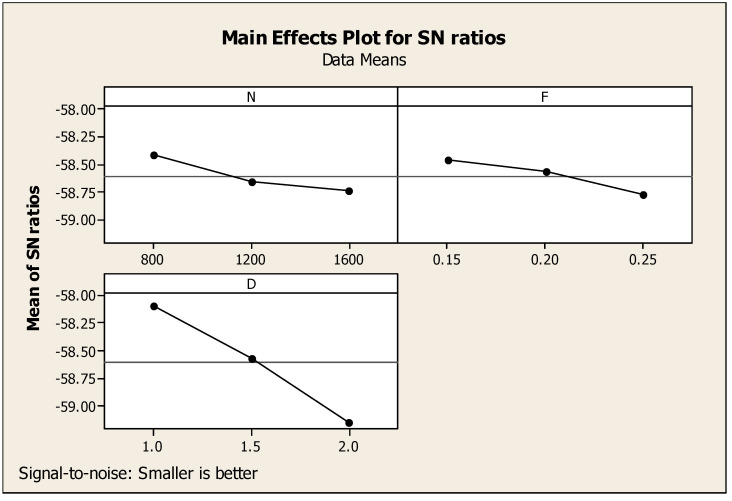
CF major effects diagram with respect to signal-to-noise ratios.

**Figure 10:**
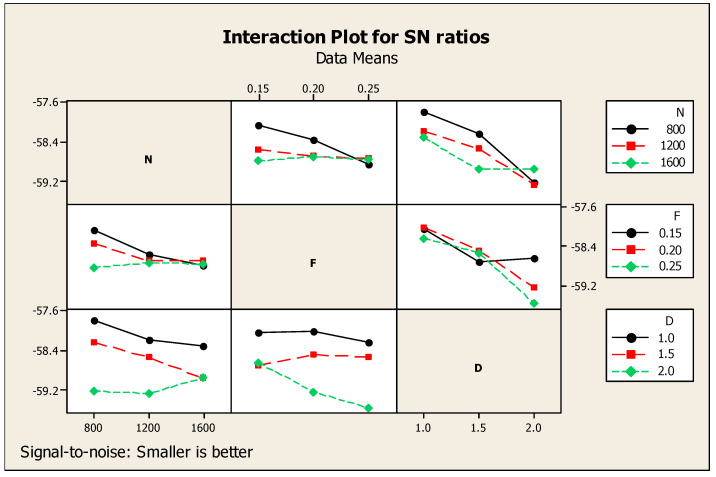
CF interaction diagram.

With respect to CF’s major effects on SNR, the cutting force alone was considered for the observation. In this regard, Table 6 highlights the Taguchi analysis details of CF versus N, F, and D, and it also provided subsequent ranking i.e., first, second, and third for cutting depth, speed, and feed, respectively. Figure 9 shows that for the ratio of SN with regard to the response such as cutting force, smaller is a better standard condition. Similarly, Figure 10 shows the interaction diagram for the ratio of SN with regard to the cutting force plotted under the smaller is better condition. Figure 10 provides the response information using the combination of two parameters such as N, F, and D. The combination of two parameters enlighten the response of cutting force in a detailed manner. Above, Figure 9 and Figure 10 show that the lowest cutting force among the results was achieved at 1600 rpm of cutting speed, 0.25 mm/min of feed rate, and 2.0 mm of cutting depth.

**Figure 11:**
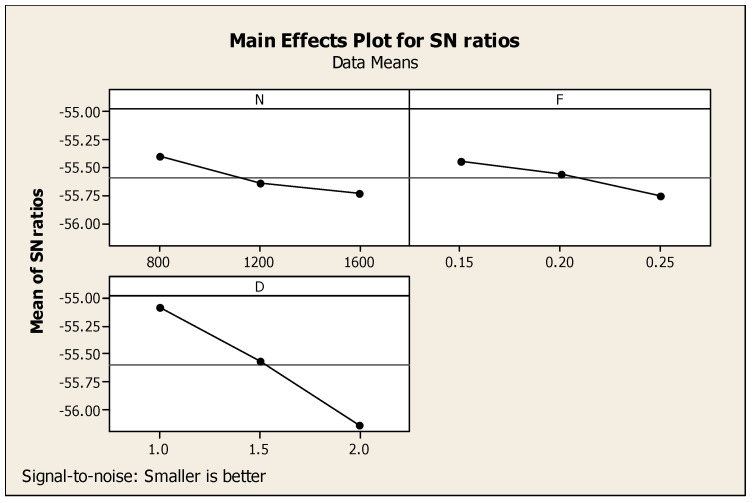
SR and CF-related major effects diagram with respect to signal-to-noise ratios.

**Figure 12:**
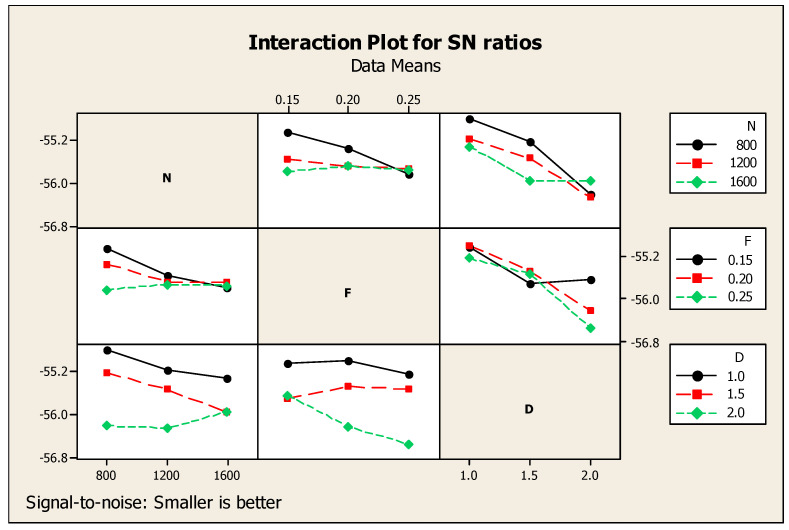
SR and CF-related interaction diagram.

**Figure 13:**
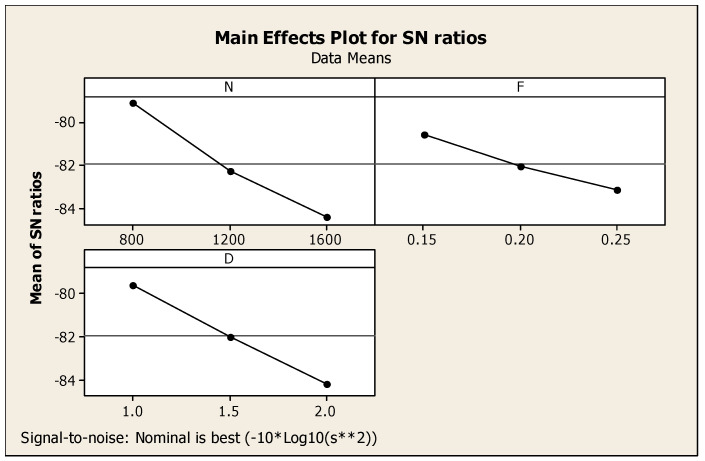
MRR, SR, and CF-related main effects diagram with respect to signal-to-noise ratios.

**Figure 14:**
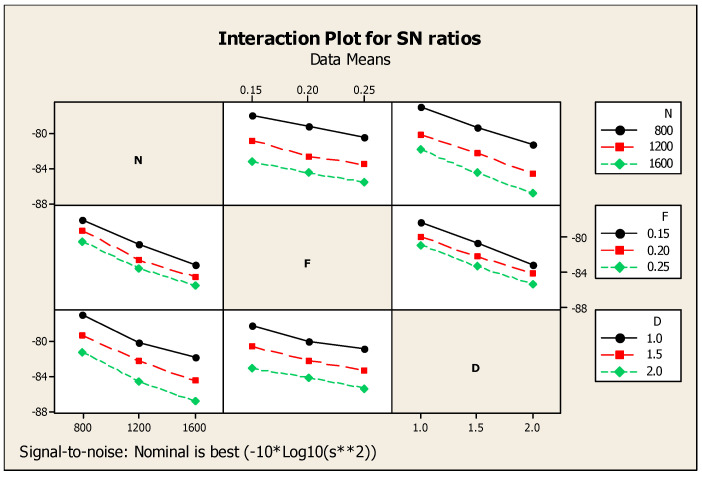
MRR, SR, and CF-related interaction diagram.

It is worth noting that each response was related with process parameters whereby Taguchi analysis detailed out two responses such as SR and CF versus process parameters such as N, F, and D, as mentioned in Table 7, with subsequent ranking order. Cutting depth obtained first rank, speed was placed second, and feed was ranked third. Figure 11 eloquently shows the major effects for ratio of SN with respect to SR and CF. Similarly, Figure 12 shows the consequent interaction diagram for ratios of SN for SR and CF with a minor is better condition, because both the SR and CF preferable values were set to be minimum. The same figure also showed the relation of the various factors such as N, F, and D as the separate diagrams of the combination with the responses such as CF and SR in a single diagram. For these parameters, minimum CF and SR responses were achieved at 1600 rpm of speed, 0.25 mm per min of feed, and 2.0 mm of cutting depth. After comparison of one and two responses with respect to three parameters, three responses such as MRR and SR with CF versus process parameters such as N, F, D of Taguchi analysis details are mentioned in Table 8 with ranking. For the three responses, the speed reached the first rank, second rank is obtained by cutting depth, and then, the third is the feed under the nominal is best condition. Major effects regarding the ratio of SN with respect to the responses such as SR and MRR with CF, and an interaction diagram for the ratio of SN with respect to MRR, SR, and CF are clearly mentioned in Figure 11 and Figure 12 in subsequent order. Moreover, Figure 13 gives a comparison of the individual factors such as N, F, and D as separate diagrams with all responses such as SR and MRR with CF. In this regard, under the nominal is best condition, all responses attained 800 rpm of speed, 0.15 mm/min of feed, and 2.0 mm of depth of cut. Nonetheless, these specs were selected as optimum values. In addition, Figure 14 shows the MRR, SR, and CF-related interaction diagram for ratios on SN for MRR, SR, and CF with the nominal is best condition.

**Figure 15:**
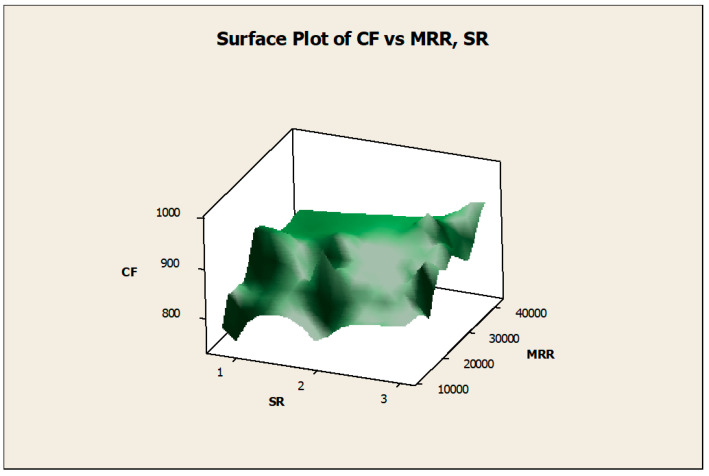
Surface diagram of CF with respect to MRR and SR.

**Figure 16:**
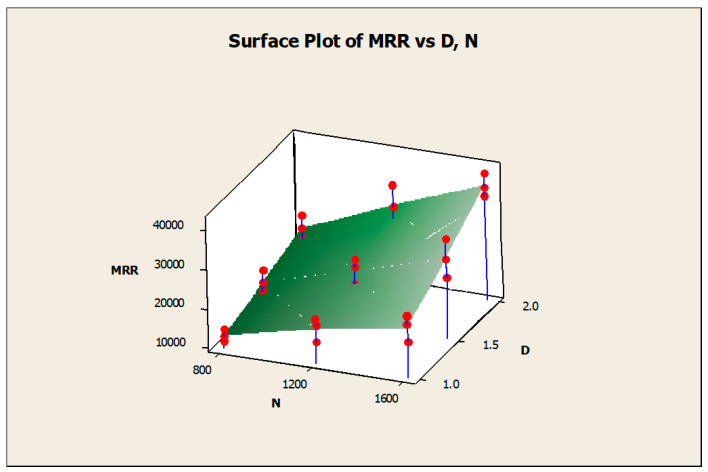
Surface diagram of MRR with respect to N and D.

**Figure 17:**
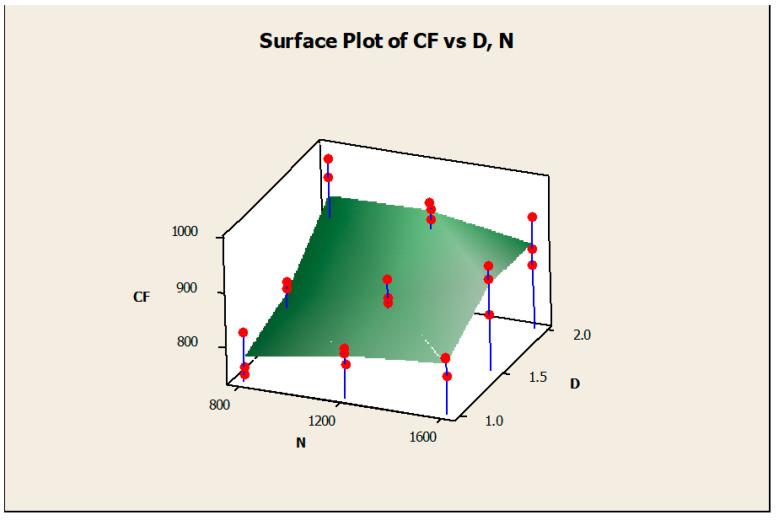
Surface diagram of CF with respect to N and D.

**Figure 18:**
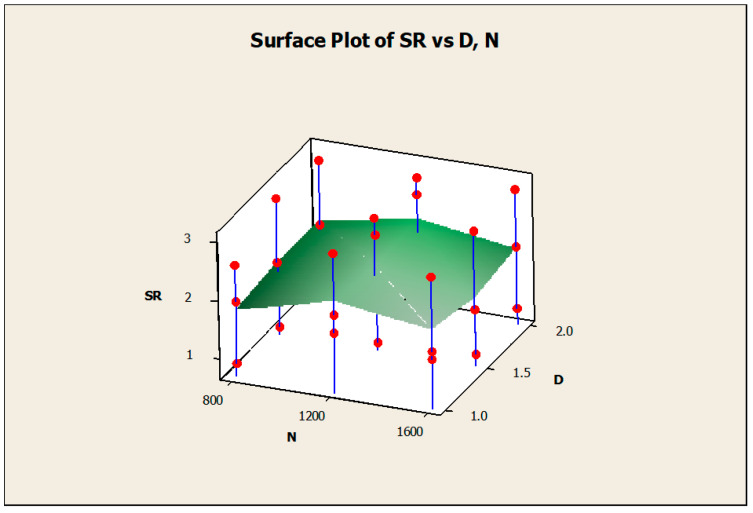
Surface diagram of SR with respect to N and D.

The surface diagrams helped to clearly identify the response behavior on the basis of the various input factors considered. Figure 15 gives the surface diagram of the response CF with respect to another two responses such as MRR and SR. The X-axis belongs to SR, the Y-axis belongs to CF, and the Z-axis belongs to MRR. The variations of the experimental results were plotted as the surface diagram. The maximum and minimum values were mentioned as the highest and lowest points in the three-dimensional graphical representation. Similarly, Figure 16 clearly mentions the surface diagram of the material removal rate relating to N (X-axis) and D (Z-axis). The various points of interest were clearly demonstrated with the red color points on the surface. Figure 17 demonstrated the surface diagram of cutting force with regard to the N (X-axis) and D (Z-axis). Similar to Figure 17, it becomes evident from Figure 18 to point out the surface diagram of surface roughness with respect to N (X-axis) and D (Z-axis).

**Figure 19:**
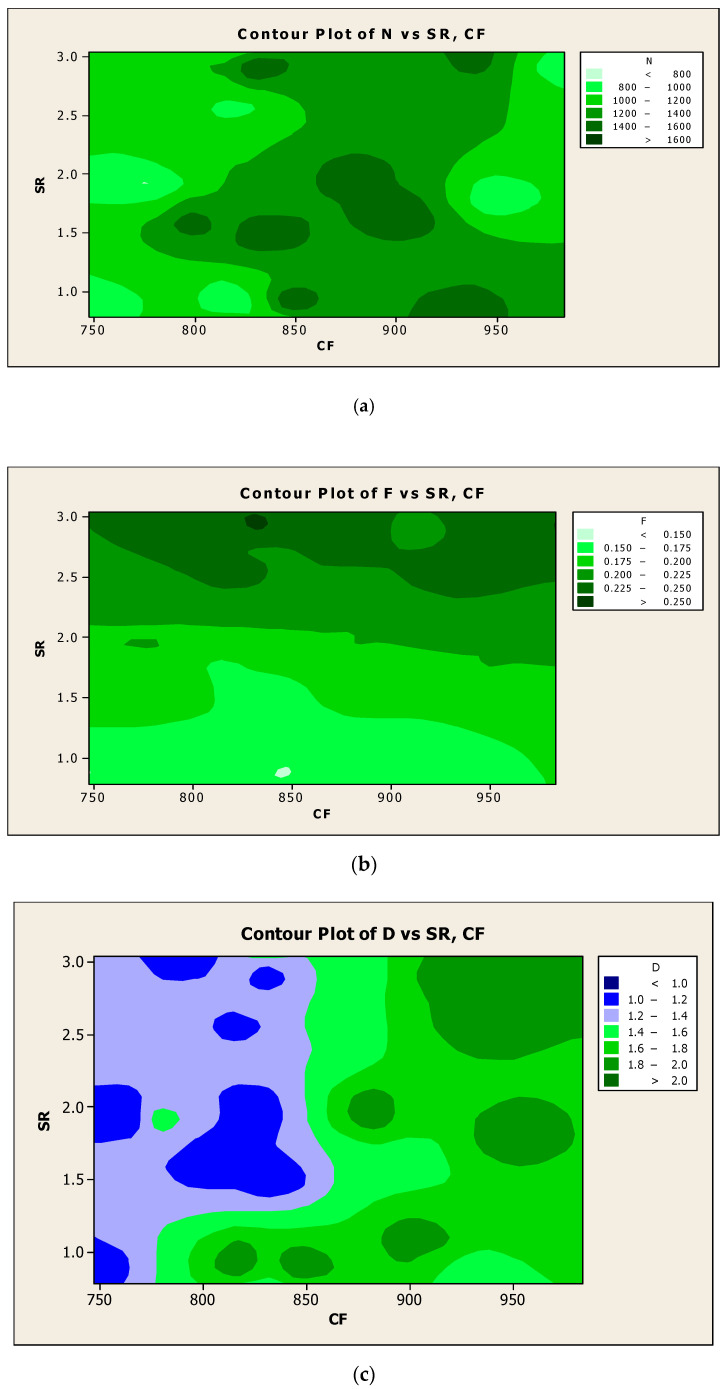
Contour diagrams with respect to SR and CF for (**a**) N, (**b**) F, and (**c**) D.

**Figure 20:**
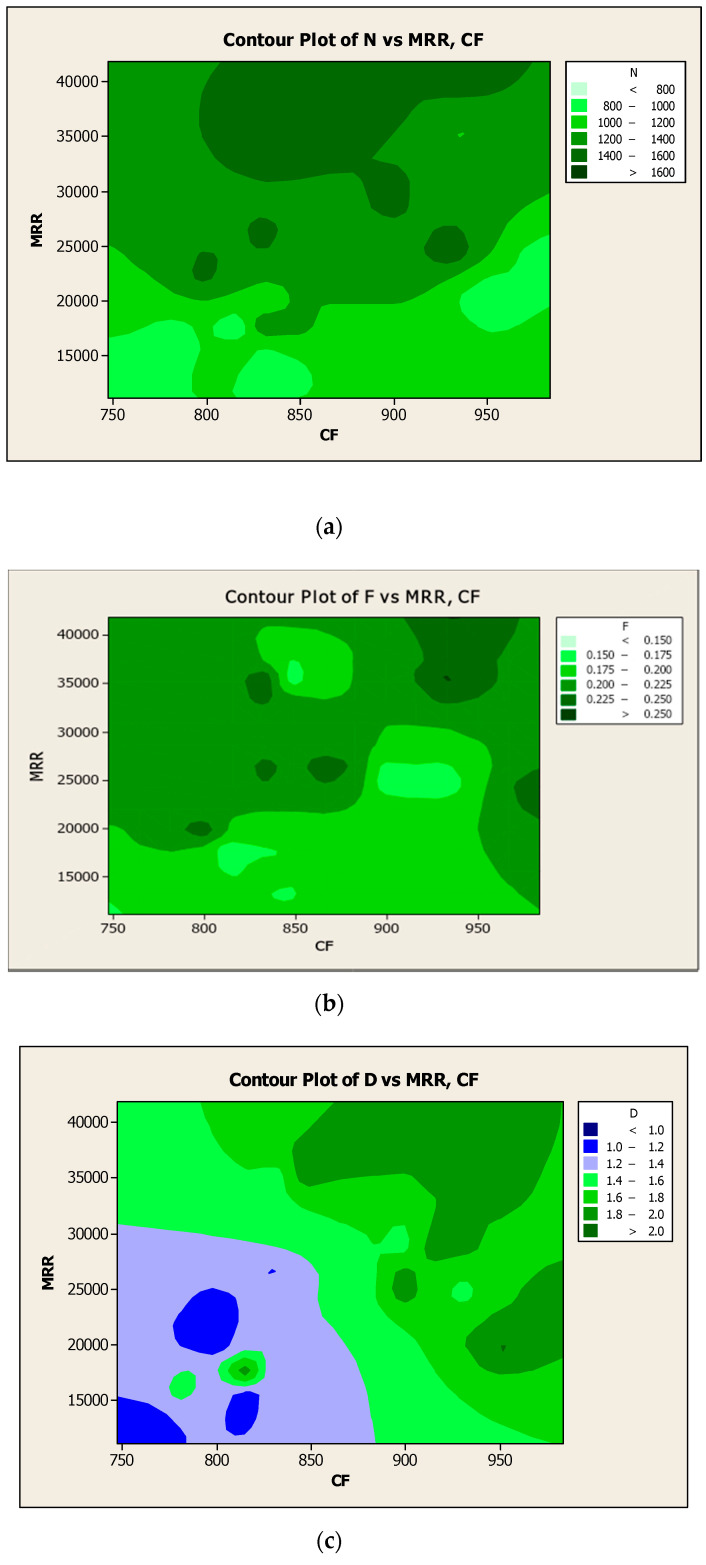
Contour diagrams with respect to MRR and CF for (**a**) N, (**b**) F, and (**c**) D.

The experimental results of the responses such as SR and CF were plotted as contour diagrams with respect to the process parameters such as N, F, and D, respectively, as shown in Figure 19a–c. Results were observed in terms of variation of color in the contour surface, which clearly expressed response results with regard to the influencing factors. Similarly, the investigational results of the responses such as MRR and CF were represented as contour diagrams with respect to the process parameters such as N, F, and D respectively, as shown in Figure 20a–c. The differences in outcomes were highlighted as the different color intensity in the contour surface, which was visible in the response results with respect to the influencing factors. 

**Figure 21:**
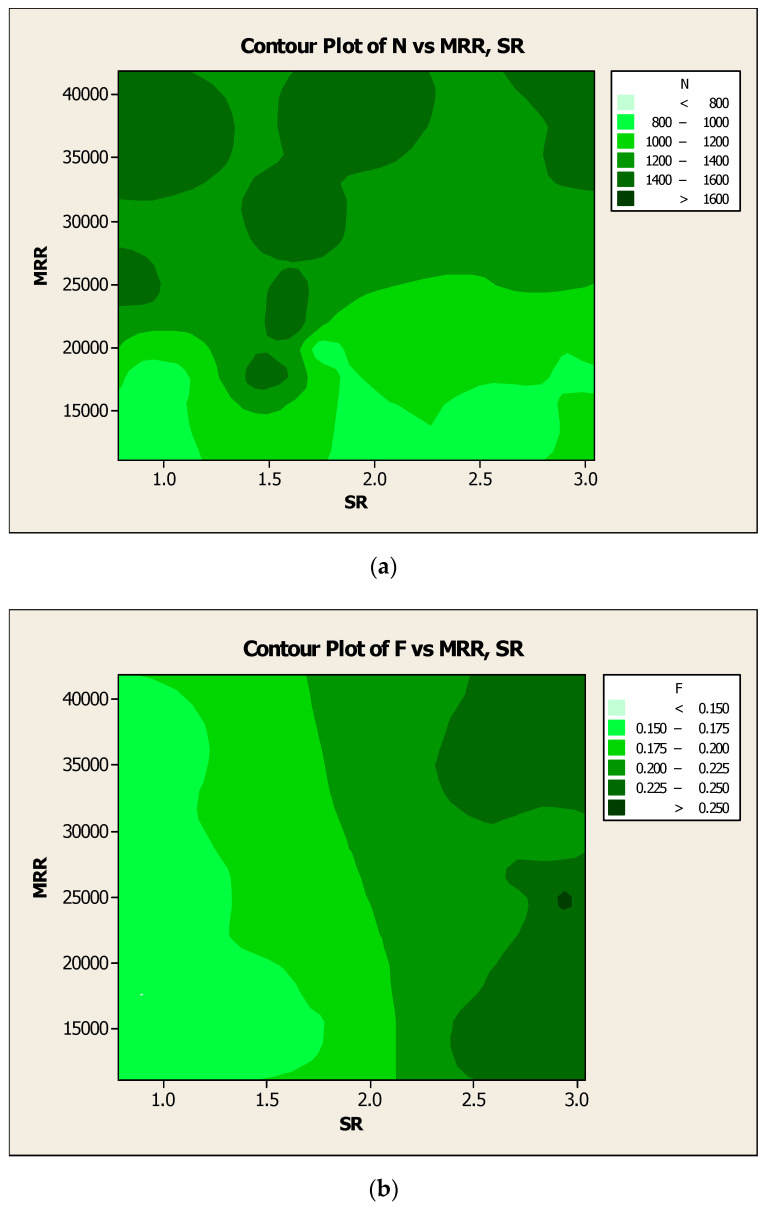

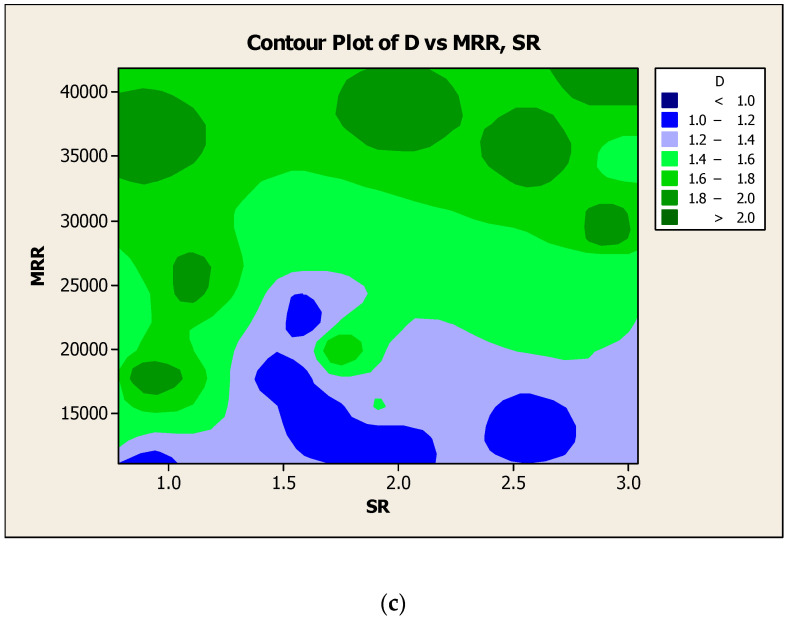
Contour diagrams with respect to MRR and SR for (**a**) N, (**b**) F, and (**c**) D.

Likewise, the trial results of the responses such as MRR and SR were symbolized as contour diagrams with respect to the process parameters such as N, F, and D respectively in Figure 21a–c. There were differences in the outcomes, which were pointed out as different color intensities on the contour surface, which in turn visibly communicated the disparity in the response results with respect to the influencing factors. The variations in the surface with various color intensities to understand the impact on the responses is based on the turning process parameters.

**Figure 22:**
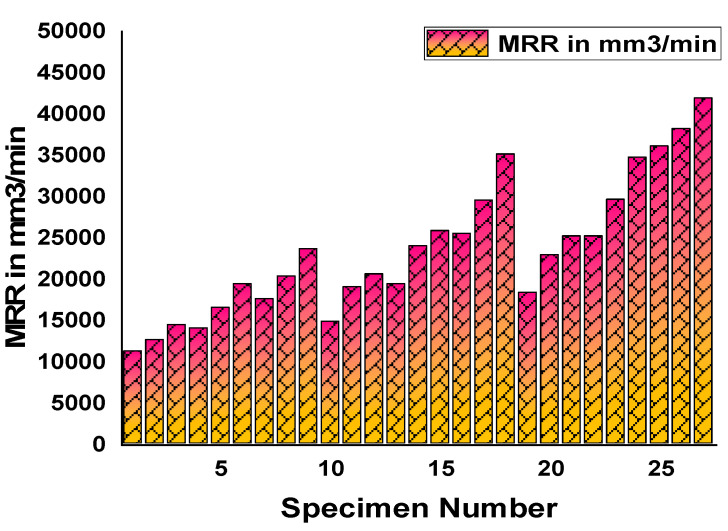
Material removal rates (mm^3^/min) for each specimen.

**Figure 23:**
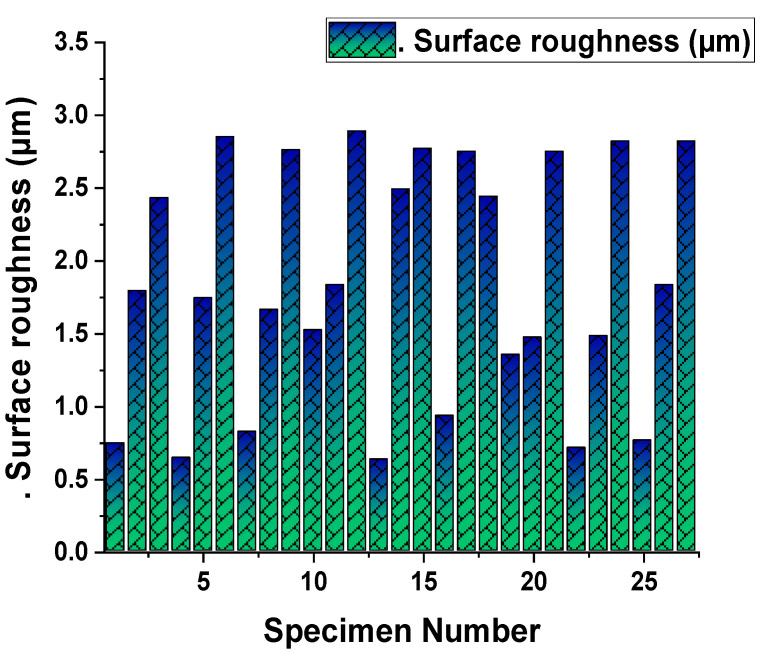
Surface roughness (µm) for each specimen.

**Figure 24:**
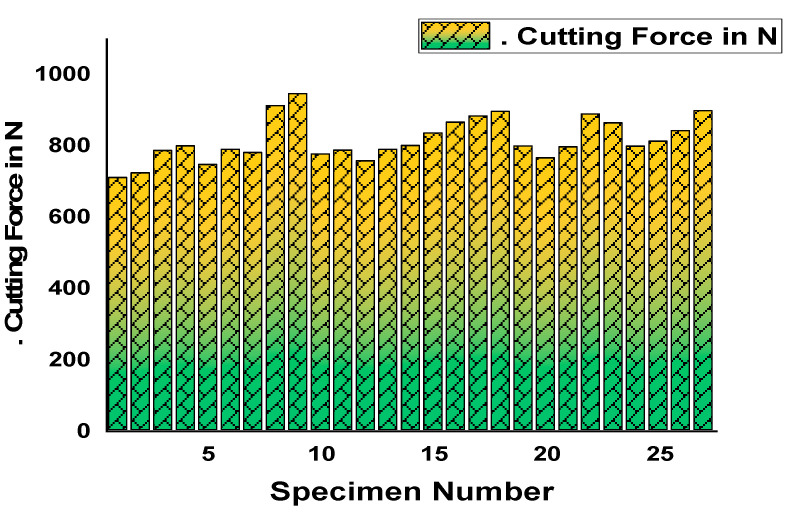
Cutting force (N) for each specimen.

Figure 22 shows a graphical representation of the experimentally measured results of the material removal rate (MRR) in mm^3^/min (Y-axis) for each specimen from 1 to 27 (X-axis). In this aspect, MRR fluctuated from maximum to minimum and vice versa. At last, the maximum MRR was obtained for the 27^th^ specimen, and the minimum MRR was obtained by the first specimen. Figure 23 plots the experimentally measured results of surface roughness in µm (Y-axis) for the entire specimen considered from 1 to 27 (X-axis). SR had heavy fluctuations as per the results as evident by the sine waves. In addition, maximum and minimum values were reached in between successive results. Similarly, Figure 24 was plotted to demonstrate the cutting force in N (Y-axis) for each specimen from 1 to 27 (X-axis).

**Figure 25:**
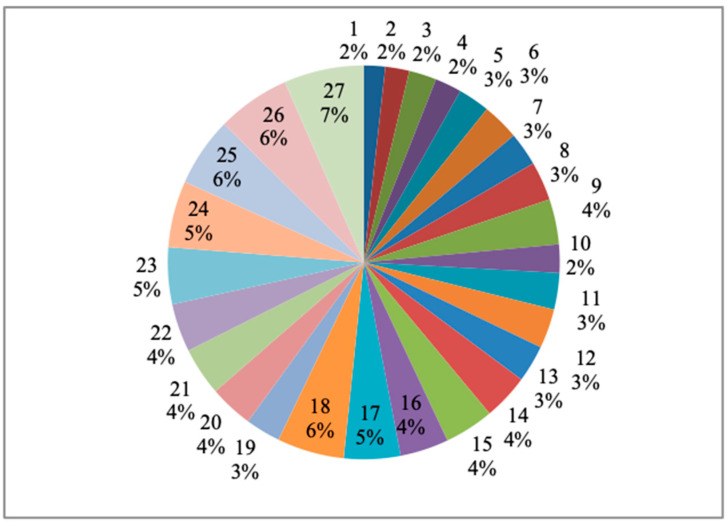
Process parameters influence on the material removal rate (mm^3^/min) for each specimen.

**Figure 26:**
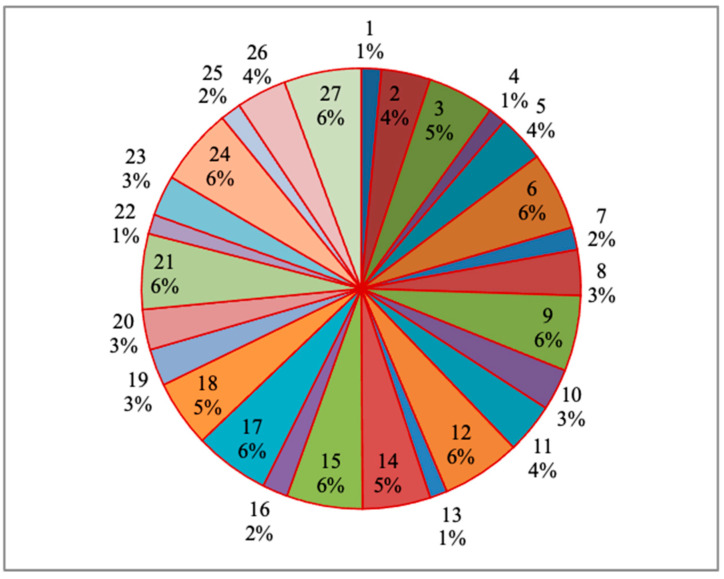
Process parameters influence on the surface roughness (µm) for each specimen.

Figure 25 represent the process parameters’ influence on the response, particularly for the material removal rate (mm^3^/min) for each specimen on the basis of the percentage of the total contribution. Here, the maximum influence percentage is 7%, which is covered by the 27th specimen. Similarly, the least percentage is 2%, and in this regard, more than five specimens reached these values among all the specimens. Figure 26 showed the process parameters’ influence in percentage variation for the response such as the surface roughness (µm) for each specimen. In this regard, the response maximum contribution is 6%, which was held by eight specimens, and the least contribution was 1%, which was held by four specimens amongst all specimens.

**Figure 27:**
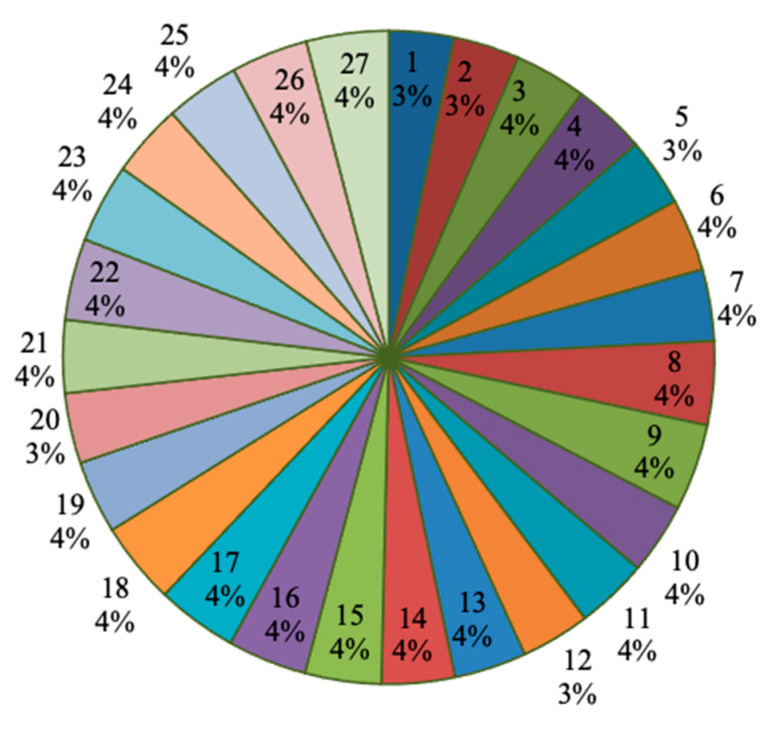
Process parameters influence on the cutting force (N) for each specimen.

Similarly, Figure 27 expresses the response as the cutting force (N) with respect to the process parameters in percentage of contribution. In this aspect, the maximum contribution was 4%, and the minimum contribution was 3%. There were only two percentage variations visible among all the specimens. Only five specimens had 3% of contribution, and the remaining twenty-two specimens had 4% of contribution.

**Figure 28:**
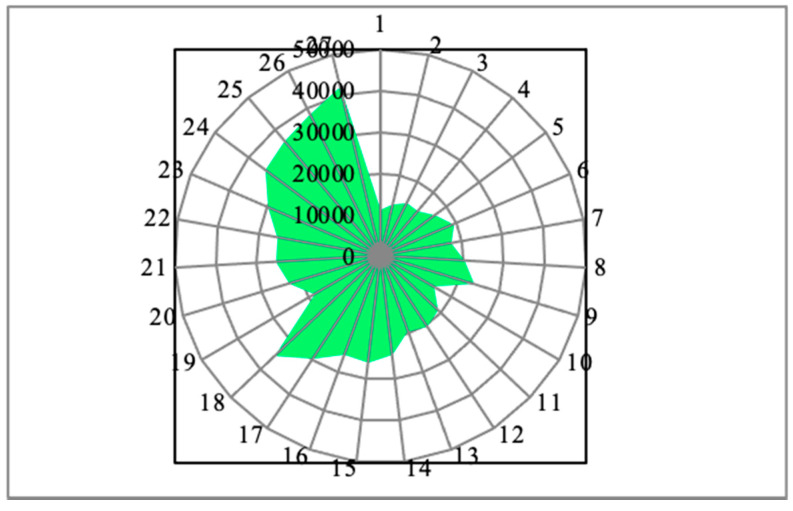
Radar diagram of the response material removal rate (mm^3^/min) for each specimen.

**Figure 29:**
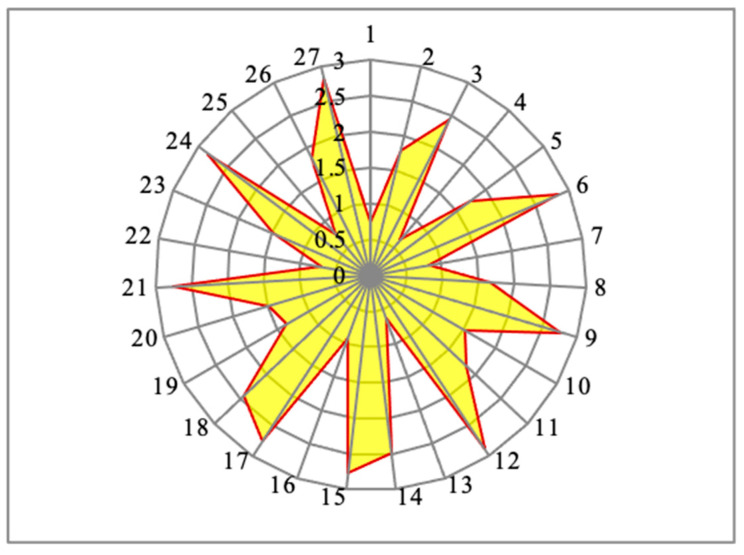
Outcomes of surface roughness (µm) for each specimen in a radar diagram.

The experimental results of each specimen’s MRR values were plotted as a radar diagram with the color of light green, as shown in Figure 28. It contains the radius lines as the representation of the individual specimens, and the different concentric circles represent the values of the material removal rate. Similarly, Figure 29 clearly demonstrates the experimental outcomes of the response such as surface roughness as a radar diagram. In this radar diagram, the specimen’s numbers and the corresponding surface roughness values were represented by the radial lines and the concentric circles, respectively. From this aspect, the shape of the surface roughness values relation came out to be like a star shape without uniform intervals, as shown in the color yellow.

**Figure 30:**
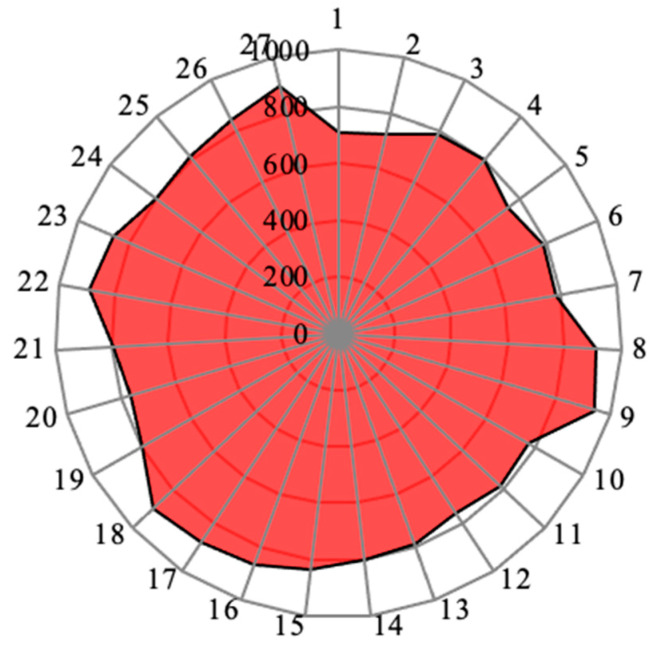
Cutting force (N) results for each specimen as radar diagram.

Figure 30 represents the experimental results of the cutting force as a graphical representation of the radar diagram. In this, concentric circles are the representations of the cutting forces variations. Then, the radial lines represent the individual specimens. Here, the shape came out to be almost a circle cut in the outer surface lines. The maximum cutting forces reached were more than 800 N. Specimens 9, 8, and 27 reached the maximum values among all the specimens’ values.

**Figure 31:**
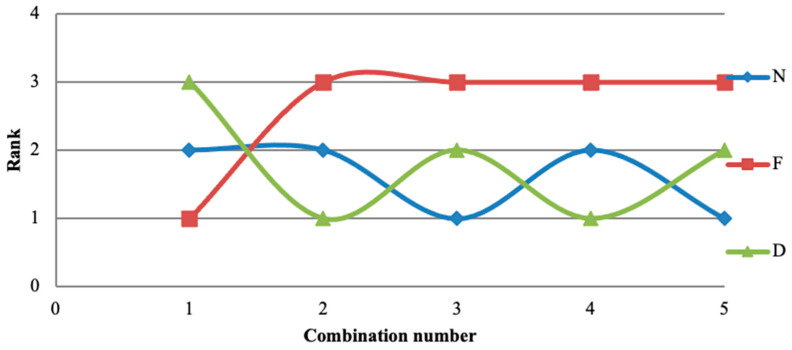
Ranking of process parameters with respect to various combinations in Table 9.

Table 9 mentions the various rankings of the process parameters based on five various combinations of responses such as MRR standalone, SR standalone, CF standalone, combination of SR and CF, and the last combination is MRR, SR, and CF. The corresponding graphical representation is shown in Figure 31. Among these five combinations of output, feed reached the third position in four combinations. So, only the remaining two were considered, and the results were compared with specimen parameters. Amongst these, two have fluctuations in each position. Nonetheless, the obtained values also help to assess the process parameters influence on the all the experimental testing conditions to identify the suitable one.

## 4. Conclusions

The proposed work takes into consideration the optimization of speed of cutting, feed, and cutting depth on the turning of Aluminum 7075 alloy through a CNC machine. When examining their effects on the metal removal rate, roughness of the machined surface, and force of cutting with the assist of L27 array, the Taguchi method produced substantial conclusions by taking into account the SN ratio, various surface diagrams, contour diagrams, and ranking positions.

The maximum material removal rate was obtained at a speed of 1600 rpm, and a 0.25 mm/min feed with 2 mm cutting depth was preferred for the specimen number 27. Moreover, the least amount of surface roughness was reached through a speed of 1200 rpm and a 0.25 mm/min feed with 1 mm cutting depth for specimen 12. The lowest possible cutting force was accomplished at a speed of 1600 rpm and 0.25 mm/min feed with 2 mm cutting depth for specimen 27. Both parameters, i.e., CF and SR, achieved the smallest amount at 1600 rpm speed, 0.25 mm/min feed, and 2.0 mm depth of cut for specimen 27. By taking the nominal is best condition with respect to three responses, we attained 800 rpm speed, 0.15 mm/min feed and 2.0 mm depth of cut in specimen 7. Nonetheless, specimen 27 demonstrated a speed of 1600 rpm, and a 0.25 mm/min feed with 2 mm cutting depth were chosen as the optimum limitations for the greatest MRR and least amount of SR and CF.

## Figures and Tables

**Table 1:** CNC machine specifications with parameter for turning process and responses for the experiment.

CNC machine specifications (heavy-duty variant of std TL-160)	**Model**	**ATL 160**		
	Chuck Size	165 mm		
	Tail Stock	Hydraulic		
	Spindle Bore	25.5 mm		
	Spindle Power	3.5/5.5 KW		
	Control	FANUC Series 0i mate.		
Turning Process Parameters	Speed (N) in rpm	800	1200	1600
	Feed (F) in mm per rev	0.150	0.200	0.250
	Depth of cut (D) in mm	1.00	1.50	2.00
Responses	MRR—Metal removal rate in mm^3^/min			
	SR—Roughness on machined surface in µm			
	CF—Force of cutting in N			

**Table 2:** Summary of responses and its best conditions.

Comparison	Responses	Better/Best Conditions
1	Material Removal Rate (MRR)	Larger (High)
2	Surface Roughness (SR)	Smaller
3	Cutting Force (CF)	Smaller
4	Surface Roughness (SR) and Cutting Force (CF)	Smaller
5	Material Removal Rate (MRR), Surface Roughness (SR) and Cutting Force (CF)	Nominal is best

**Table 3:** L27 orthogonal array with experimental results of responses.

Specimen Number	Process Parameters			Responses		
	N in Revolutions per Minute	F in mm per Revolution	D in mm	MRR in mm^3^ per Minute	SR in µm	CF in Newton
1	800	0.15	1	11,194.8	0.74	710.12
2	800	0.2	1	12,594.15	1.79	723.22
3	800	0.25	1	14,393.32	2.43	786.23
4	800	0.15	1.5	13,993.5	0.64	799.56
5	800	0.2	1.5	16,516.92	1.74	746.56
6	800	0.25	1.5	19,375.62	2.85	789.31
7	800	0.15	2	17,563.52	0.82	780.45
8	800	0.2	2	20,307.82	1.66	912.32
9	800	0.25	2	23,630.92	2.76	946.31
10	1200	0.15	1	14,816.65	1.52	776.32
11	1200	0.2	1	19,010.04	1.83	786.98
12	1200	0.25	1	20,561.88	2.89	756.84
13	1200	0.15	1.5	19,375.62	0.63	789.56
14	1200	0.2	1.5	23,988.86	2.49	800.12
15	1200	0.25	1.5	25,834.16	2.77	835.35
16	1200	0.15	2	25,484.32	0.93	865.66
17	1200	0.2	2	29,538.65	2.75	883.25
18	1200	0.25	2	35,127.04	2.44	896.21
19	1600	0.15	1	18,318.77	1.35	798.91
20	1600	0.2	1	22,898.46	1.47	765.54
21	1600	0.25	1	25,188.31	2.75	796.46
22	1600	0.15	1.5	25,188.31	0.71	888.65
23	1600	0.2	1.5	29,633.3	1.48	864.26
24	1600	0.25	1.5	34,742.49	2.82	798.65
25	1600	0.15	2	36,102.79	0.76	812.12
26	1600	0.2	2	38,226.48	1.83	841.73
27	1600	0.25	2	41,925.82	2.82	897.87

**Table 4:** Taguchi analysis details of MRR vs. speed (N), feed (F), and depth of cut (D).

SN Ratios Response Table			
Levels	N	F	D
1	84.13	85.56	84.60
2	87.22	87.00	86.95
3	89.30	88.08	89.10
Delta	5.17	2.52	4.50
Rank	1	3	2

**Table 5:** Taguchi analysis details of SR vs. N, F, and D.

SN Ratios Response Table			
Levels	N	F	D
1	−4.4630	−0.1148	−5.5535
2	−5.9788	−6.0466	−4.5550
3	−4.8798	−9.1602	−5.2132
Delta	1.5158	9.0454	0.9985
Rank	2	1	3

**Table 6:** Taguchi investigation details of CF vs. N, F, and D.

SN Ratios-Based Response Table			
Level	N	F	D
1	−58.41	−58.46	−58.10
2	−58.66	−58.57	−58.57
3	−58.74	−58.78	−59.15
Delta	0.33	0.31	1.05
Rank	2	3	1

**Table 7:** Taguchi investigation details of SR and CF vs. N, F, and D.

SN Ratios Response Table			
Level	N	F	D
1	−55.40	−55.45	−55.09
2	−55.65	−55.56	−55.56
3	−55.73	−55.77	−56.14
Delta	0.33	0.31	1.05
Rank	2	3	1

**Table 8:** Taguchi investigation details of MRR, SR, and CF vs. N, F, and D.

SN Ratios Response Table			
Level	N	F	D
1	−79.14	−80.60	−79.62
2	−82.28	−82.06	−82.01
3	−84.40	−83.16	−84.19
Delta	5.26	2.56	4.57
Rank	1	3	2

**Table 9:** Ranking of various combinations of responses.

Comparison	Responses	Conditions Better/Best	N	F	D
1	MRR	Larger	2	1	3
2	SR	Smaller	2	3	1
3	CF	Smaller	1	3	2
4	SR and CF	Smaller	2	3	1
5	MRR, SR, and CF	Nominal is best	1	3	2

## Data Availability

The dataset can be requested from the corresponding authors upon a formal request.

## References

[1] Rajmohan T., Palanikumar K., Prakash S. (2013). Grey-fuzzy algorithm to optimise machining parameters in drilling of hybrid metal matrix composites. Compos. Part B Eng..

[2] Kılıçkap E., Çakır O., Aksoy M., Inan A. (2005). Study of tool wear and surface roughness in machining of homogenised SiC-p reinforced aluminium metal matrix composite. J. Mater. Process. Technol..

[3] Grzesik W. (2008). Influence of tool wear on surface roughness in hard turning using differently shaped ceramic tools. Wear.

[4] Aouici H., Yallese M.A., Chaoui K., Mabrouki T., Rigal J.-F. (2012). Analysis of surface roughness and cutting force components in hard turning with CBN tool: Prediction model and cutting conditions optimization. Measurement.

[5] Kulandaivel A., Kumar S. (2020). Effect of magneto rheological minimum quantity lubrication on machinability, wettability and tribological behavior in turning of Monel K500 alloy. Mach. Sci. Technol..

[6] Sathish T., Sevvel P., Sudharsan P., Vijayan V. (2021). Investigation and optimization of laser welding process parameters for AA7068 aluminium alloy butt joint. Mater. Today Proc..

[7] Ross P.J. (1988). Taguchi Techniques for Quality Engineering.

[8] Elsayed E., Chen A. (1993). Optimal levels of process parameters for products with multiple characteristics. Int. J. Prod. Res..

[9] Sathish T., Karthick S. (2020). Wear behaviour analysis on Aluminium Alloy 7050 with Reinforced SiC through Taguchi approach. J. Mater. Res. Technol..

[10] Ranganath M.S., Vipin H. (2013). Optimization of Process Parameters in Turning Operation Using Taguchi Method and Anova: A Review. Int. J. Emerg. Technol. Adv. Eng..

[11] Mandal K., Kuar A.S., Mitra S. (2018). Experimental investigation on laser micro-machining of Al 7075 Alloy. Opt. Laser Technol..

[12] Karabulut S. (2015). Optimization of surface roughness and cutting force during AA7039/Al2O3 metal matrix composites milling using neural networks and Taguchi method. Measurement.

[13] Muñoz-Escalona P., Maropoulos P.G. (2010). Artificial neural networks for surface roughness prediction when face milling Al 7075–T7351. J. Mater. Eng. Perform..

[14] Ajithkumar J.P., Xavior M.A. (2017). Cutting Force and Surface Roughness Analysis During Turning of Al 7075 Based Hybrid Composites. Procedia Eng..

[15] Azizi M.W., Belhadi S., Yallese M.A., Mabrouki T., Rigal J.-F. (2012). Surface roughness and cutting forces modeling for optimization of machining condition in finish hard turning of AISI 52100 steel. J. Mech. Sci. Technol..

[16] Tzeng C.-J., Lin Y.-H., Yang Y.-K., Jeng M.-C. (2009). Optimization of turning operations with multiple performance characteristics using the Taguchi method and Grey relational analysis. J. Mater. Process. Technol..

[17] Sathish T., Sabarirajan N., Karthick S. (2020). Machining parameters optimization of Aluminium Alloy 6063 with reinforcement of SiC composites. Mater. Today Proc..

[18] Chen D.-C., Chen C.-F. (2007). Use of Taguchi method to study a robust design for the sectioned beams curvature during rolling. J. Mater. Process. Technol..

[19] Lin C.L. (2004). Use of the Taguchi Method and Grey Relational Analysis to Optimize Turning Operations with Multiple Performance Characteristics. Mater. Manuf. Process..

[20] Lavanya K.M., Suresh R.K., Priya A.S.K., Reddy V.D. (2013). Optimization of process parameters in turning operation of AISI-1016 alloy steels with CBN using Taguchi method and ANOVA. IOSR J. Mech. Civ. Eng..

[21] Sathish T. (2019). Experimental investigation of machined hole and optimization of machining parameters using electrochemical machining. J. Mater. Res. Technol..

[22] Verma N.K., Sikarwar A.S. (2015). Optimizing Turning Process by Taguchi Method Under Various Machining Parameters. Int. J. Eng. Res. Technol..

[23] Jha S.K. (2016). Parametric optimization of turning process using Taguchi method and ANOVA analysis. Int. J. Adv. Eng. Technol..

[24] Rudrapati R., Sahoo P., Bandyopadhyay A. (2016). Optimization of process parameters in CNC turning of aluminium alloy using hybrid RSM cum TLBO approach. IOP Conf. Ser. Mater. Sci. Eng..

[25] Khorasani A.M., Yazdi M.R.S., Safizadeh M.S. (2011). Tool Life Prediction in Face Milling Machining of 7075 Al by Using Artificial Neural Networks (ANN) and Taguchi Design of Experiment (DOE). Int. J. Eng. Technol..

[26] Sathish T. (2019). Optimization of Co 2 laser cutting parameters on Austenite stainless steel using Gray Relational Analysis. Int. J. Mech. Eng. Technol..

